# Endoscopic-assisted low-temperature plasma resection of tongue root schwannoma via oral approach: a case report and literature review

**DOI:** 10.3389/fped.2026.1747673

**Published:** 2026-03-31

**Authors:** Jing He, Xintao Cai, Huajun Feng, Yilin Bao, Gang Qin

**Affiliations:** Department of Otolaryngology Head and Neck Surgery, The Afﬁliated Hospital of Southwest Medical University, Luzhou, China

**Keywords:** endoscope, low-temperature plasma, oral approach, schwannoma, tongue root

## Abstract

This article reports a case of a 17-year-old male pediatric patient whose imaging and fiberoptic nasopharyngoscopy examinations initially suggested an oropharyngeal malignant tumor. The patient underwent endoscopic-assisted low-temperature plasma surgical excision of the tongue root schwannoma via oral approach, a minimally invasive method well-suited for pediatric patients, and achieved excellent therapeutic outcomes with no postoperative complications or tumor recurrence. This case provides critical clinical reference for the diagnosis and treatment of tongue root schwannomas in children and adolescents, addressing an unmet need in pediatric head and neck care.

## 1 Introduction

Schwannoma is a benign tumor derived from Schwann cells and is associated with genetic syndromes such as schwannomatosis or neurofibromatosis type 2 (NF2) (1). The pathogenesis may be caused by the inactivation of the Nf2 tumor suppressor gene on chromosome 22 (2–5). Schwannomas commonly occur in individuals aged 20–40 years and are most frequently found in peripheral nerve trunks, particularly on the flexor surfaces of the limbs (elbows, wrists, knees, etc.), followed by the neck, face, scalp, hands, and posterior mediastinum. Schwannomas occurring in the tongue root region are relatively rare, and they often present as painless, slow-growing, solitary masses. As they enlarge, they may cause discomfort such as tongue pain and sensory abnormalities (6, 7). However, the above symptoms are usually non-specific and are mostly caused by the local compression of the tumor itself on adjacent structures.

Schwannomas in the tongue root region are often asymptomatic, and may manifest as irregular, fragile masses or slowly growing nodular lesions. Preoperative examinations often make it difficult to distinguish benign from malignant lesions, thus timely and accurate early diagnosis and treatment are often not achieved, leading to missed diagnoses or misdiagnoses (7–10). This article reports a case of endoscopic-assisted low-temperature plasma resection of a tongue root schwannoma via oral approach, combined with a literature review to analyze the clinical characteristics of this disease. It aims to provide reference experience for the diagnosis and treatment of tongue root schwannoma and to reduce missed and misdiagnosis.

## 2 Case description

**Figure 1:**
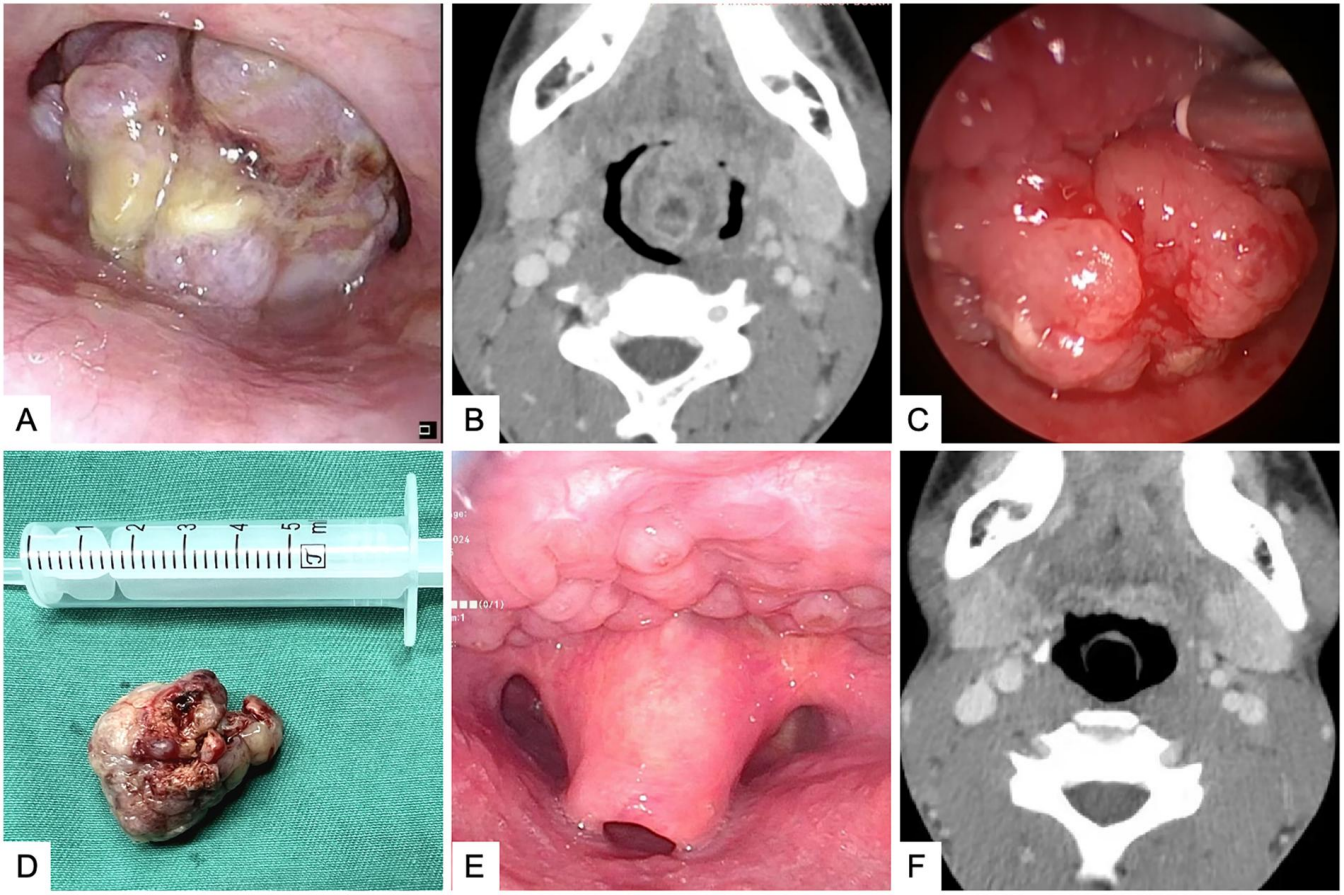
Preoperative, intraoperative, and postoperative photographs of the patient. **(A)** Preoperative fiberoptic nasopharyngoscopy revealed a nodular neoplasm with a diameter of approximately 3 cm at the midline of the tongue root, with yellow secretion on the surface and unclear boundaries; **(B)** Preoperative contrast-enhanced neck CT showed an irregular soft tissue mass approximately 3 cm × 3 cm in the oropharynx with necrosis and uneven enhancement, broadly connected to the tongue root with unclear boundaries, narrowing the oropharyngeal cavity, suggesting the possibility of a malignant tumor in the tongue root region; **(C)** Intraoperatively, the mass appeared nodular, bleeding easily upon touch, with a broad base and unclear boundaries; **(D)** Postoperative photograph of the tongue root mass showed a nodular mass approximately 3 cm in diameter, with an irregular surface accompanied by necrosis; **(E)** Two-year postoperative review with fiberoptic nasopharyngoscopy showed a smooth tongue root region with no tumor recurrence; **(F)** Two-year postoperative contrast-enhanced neck CT showed no abnormalities in the tongue root region, with no tumor recurrence.

A 17-year-old male patient was admitted with a chief complaint of “pharyngeal foreign body sensation accompanied by dysphagia for more than 2 years and recurrent hematemesis for 2 days”. Two years ago, the patient presented with pharyngeal foreign body sensation, dysphagia, cough, and expectoration of unknown cause, with no sore throat, hoarseness, or dyspnea. The patient did not seek any medical evaluation. Two days ago, recurrent hematemesis occurred with a total bleeding volume of approximately 10 mL. The bleeding stopped after resting calmly, accompanied by palpitations, without symptoms such as headache, dizziness, fatigue, chest tightness, shortness of breath, nausea, or vomiting. Physical examination: An irregular neoplasm was visible at the tongue root, with a rough surface and a small amount of blood scab attached. No enlarged lymph nodes were palpable in the neck. Fiberoptic nasopharyngoscopy examination: A nodular neoplasm of approximately 3 cm diameter was observed at the midline of the tongue root, with yellow secretion on the surface, easy bleeding upon touch, and unclear boundaries (Figure 1A); contrast-enhanced neck CT: An irregular soft tissue mass shadow of approximately 3 cm × 3 cm was seen in the oropharyngeal cavity, accompanied by necrosis, showing uneven enhancement, with unclear boundaries, considering the possibility of a malignant tumor (Figure 1B). The patient had no significant previous medical history.

**Figure 2:**
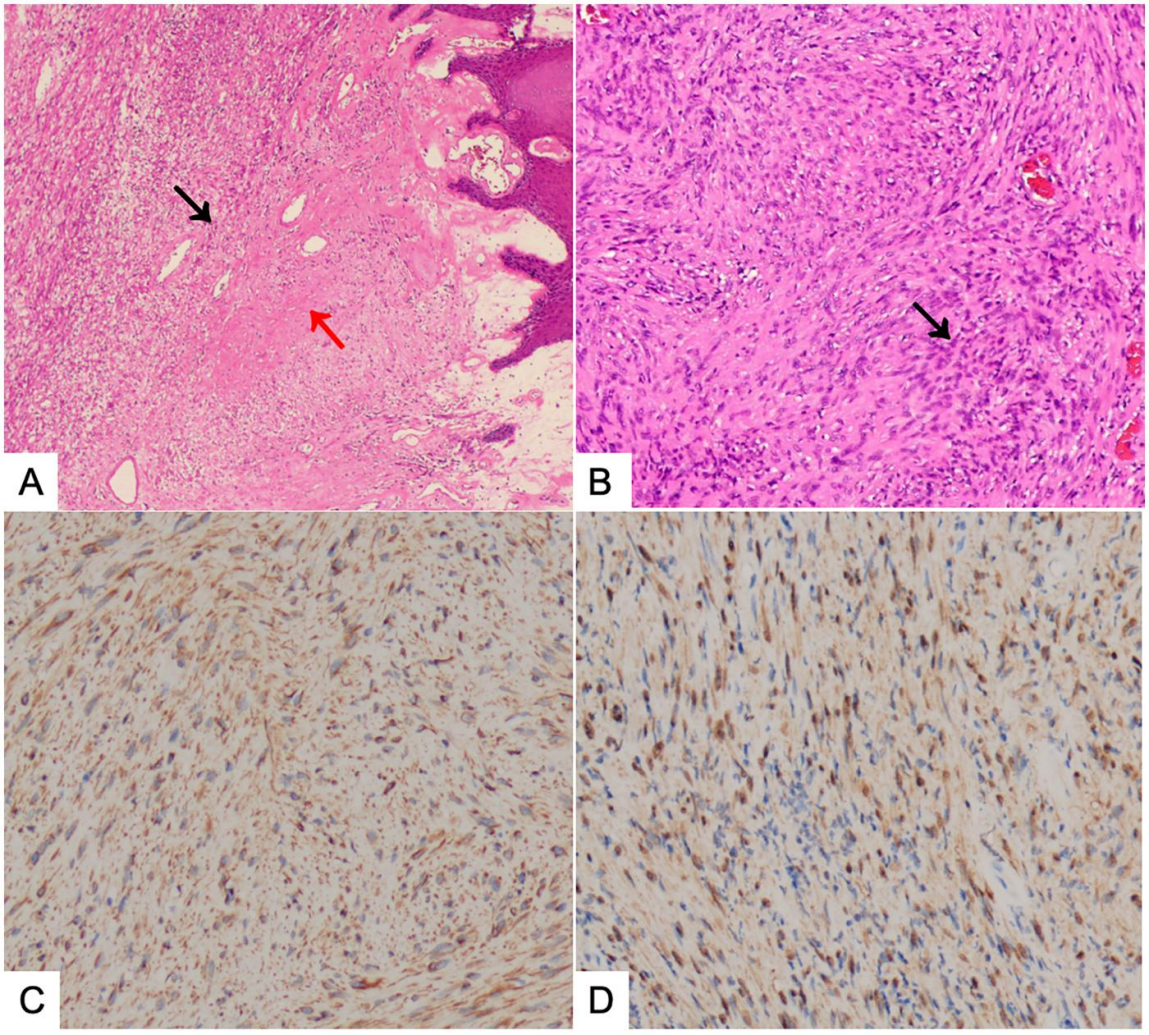
Histopathological results of the tumor post-surgery. **(A)** Microscopic photograph of Antoni A and Antoni B tissues within the Schwannoma: The left side of the field shows a high cellular density Antoni A region (black arrow), and the right side shows a loosely organized cellular Antoni B region (red arrow), forming a contrast (HE × 100); **(B)** Microscopic photograph of Antoni tissue within the Schwannoma: The image shows Verocay bodies with wavy, tightly organized nuclear palisading (black arrow) (HE × 400); **(C)** Immunohistochemistry shows characteristic Vimentin (+) for schwannoma (×100); **(D)** Immunohistochemistry shows characteristic S-100 (+) for schwannoma (×400).

Based on imaging and fiberoptic nasopharyngoscopy and laryngoscopy examination, it was considered that the preoperative diagnosis of the patient might be a malignant tumor of the tongue root region. Due to the large size of the patient's tumor, which blocked the oropharyngeal cavity, a prophylactic tracheotomy under local anesthesia was initially planned to prevent postoperative dyspnea. After general anesthesia, the area was re-disinfected and draped. Using a Boyle-Davis mouth gag (Leopard brand, H070.00), we maximally exposed the oropharyngeal cavity. With the combined use of a 4 mm diameter 30°nasal endoscope (Storz, 7240BA3D) and a high-definition camera system (Karl Storz GmbH & Co. KG, TC201 & TC302 & TM441), a broad-based exophytic nodular lesion was observed in the tongue root region, slightly offset to the left of the midline. The surface was irregular, with visible necrosis and pseudomembrane. The lesion measured approximately 3 cm in diameter and bled easily on contact. During the surgery, a biopsy of part of the tissue was taken, and the frozen section indicated a benign tumor. Therefore, an endoscopic-assisted low-temperature plasma (ArthroCare, Coblator II RF8000E) expanded resection of the tongue root mass was performed via the oral approach, and hemostasis with plasma was applied to active bleeding points. Estimated blood loss was approximately 3 mL. Postoperative histopathological diagnosis of the tumor was tongue root schwannoma, as shown in Figure 2A,B. Immunohistochemistry results indicated: Vimentin (+) (Figure 2C), S-100 (+) (Figure 2D). Postoperatively, the patient preserved normal tongue motor function, with no tongue deviation, numbness, speech disorders, bleeding, or other complications.

The tracheal tube was removed one week postoperatively. Two years after the operation, the patient showed no related discomfort. Comprehensive fiberoptic electronic nasopharyngoscopy (Figure 1E) and neck-enhanced CT examination (Figure 1F) showed no tumor recurrence.

## 3 Discussion

Schwannomas involving the oral cavity account for approximately1% of head and neck schwannomas, with tongue root localization being exceptionally rare, especially in pediatric and adolescent populations. Most schwannomas present in adults aged 20–40 years (6), this 17-year-old patient is notably younger, making this a clinically interesting and valuable addition to the limited literature on pediatric tongue root schwannomas.

Schwannoma is an uncommon benign neoplasm in the pediatric population overall. Pediatric head and neck schwannomas account for only a small proportion of pediatric head and neck tumors, and pediatric tongue schwannomas represent less than 1% of all pediatric head and neck schwannomas. Among these, tongue root schwannoma is an extremely rare subtype with an exceptionally low incidence, representing one of the rarest benign tumors in the pediatric head and neck region (11).

According to available literature, pediatric tongue root schwannoma shows no obvious gender predilection. Age at presentation mainly occurs in children and adolescents. All lesions are exclusively located in the tongue root, mostly at or close to the midline. Most patients present with pharyngeal foreign body sensation or dysphagia, while some are asymptomatic or experience recurrent oral bleeding. All reported tumors have been resected using a transoral approach, and patients generally achieve an excellent prognosis with no evidence of tumor recurrence during follow-up (12).

Pediatric tongue root schwannomas frequently present diagnostic dilemmas attributable to their nonspecific initial clinical manifestations, such as vague pharyngeal discomfort, which led to a 2-year delay in medical consultation and diagnosis in this patient. When the tumor diameter exceeds 3 cm, as observed in the present case, it may give rise to dysphagia or phonatory disturbances; furthermore, its imaging characteristics (e.g., irregular margins, heterogeneous enhancement) can mimic malignant neoplasms, thereby predisposing clinicians to preoperative misdiagnosis, a major pitfall in pediatric head and neck oncology (7). For children, preoperative biopsy or fine-needle aspiration is advisable to avoid misdirected surgical planning, safeguarding their growth and functional integrity (7). However, preoperative fine-needle aspiration was not performed in this patient due to the emergent presentation with recurrent hematemesis and the high risk of bleeding and airway obstruction caused by the hypervascular tumor. Schwannomas typically originate from the hypoglossal, lingual, or glossopharyngeal nerve, but preoperative identification of the nerve origin remains difficult (12). Schwannomas are classically characterized by radiological features of oval or spherical low-density soft-tissue masses with well-demarcated margins and non-invasive growth patterns, while contrast-enhanced computed tomography (CT) typically demonstrates either homogeneous or heterogeneous enhancement (13, 14). On MRI, schwannomas typically appear isointense on T1-weighted sequences and have heterogeneous intensity on T2-weighted sequences, usually showing bright enhancement on T1 enhanced sequences (15). Notably, the patient was admitted urgently due to recurrent hematemesis, and cervical contrast-enhanced CT was prioritized for initial evaluation, as it can quickly assess the size of the oropharyngeal tumor, the presence of internal necrosis, and potential compression on the oropharyngeal airway—critical information for urgent surgical planning. MRI, although more advantageous in displaying soft-tissue details and tumor-nerve relationships, was not performed initially due to the emergency nature of the patient's presentation. The lesion in the present pediatric patient exhibited atypical imaging findings on CT, including irregular morphology, internal necrosis, and ill-defined borders, deviations from the classic radiological profile of schwannomas that directly contributed to preoperative misdiagnosis.

Surgical excision remains the gold standard for schwannomas, and pediatric patients demand minimally invasive, low-trauma approaches to preserve tongue function and facial aesthetics. For tumors with favorable accessibility, the transoral approach is recognized as the preferred surgical strategy, as it obviates the need for cervical muscle or osseous dissection, minimizes facial-cervical scarring, and reduces the incidence of postoperative complications such as pharyngocutaneous fistula and surgical site infection, factors of paramount importance in pediatric patients, where preservation of anatomical structure, functional integrity, and cosmetic outcomes are core clinical priorities (16, 17). Nevertheless, conventional transoral surgery is often hampered by suboptimal exposure of the tongue base region and technical challenges in achieving precise hemostasis, particularly in the pediatric population with relatively smaller anatomical spaces and delicate tissue structures. With the development of transoral minimally invasive surgery, various laser systems have been widely used in tongue base tumor resection besides CO₂ laser. CO₂ laser (10,600 nm) provides high cutting precision and minimal thermal injury, serving as the gold standard for transoral resection of benign lesions such as schwannomas (18, 19). KTP laser (532 nm) shows strong absorption by hemoglobin and outstanding hemostatic performance, which is especially suitable for hypervascular tongue base lesions (20). Diode laser (810–980 nm) features deep tissue penetration and flexible fiber delivery, making it appropriate for deeply located tumors (21, 22). The selection of laser modality should be individualized based on tumor vascularity, size, and location (23).

Common clinical transoral surgical methods include oral endoscope-assisted low-temperature plasma radiofrequency ablation, laser surgery, Da Vinci robotic oral surgery, and direct surgery. Compared to traditional open surgery, transoral approaches are associated with smaller surgical wounds, lower complication rates, better functional preservation, and improved cosmetic outcomes (17). However, exposure of the tongue root region can still be limited, and hemostasis may be technically demanding under a pure transoral approach.

CO₂ laser surgery under suspension laryngoscope and microscope provides precise tissue dissection with good protection of adjacent structures, but is limited by restricted visualization of the tongue base and potential accidental thermal injury to normal mucosa (19, 24). Additionally, the exposure of the tongue root region is poor with a suspension laryngoscope. Compared to traditional oral approach surgery, the Da Vinci robotic surgery via the oral approach offers advantages such as higher anatomical efficiency and more precise surgical operations (25). However, the Da Vinci robotic surgery has drawbacks such as high acquisition and maintenance costs, leading to a heavy financial burden on patients, prolonged time for surgical site exposure, and robot positioning. These issues still require continuous improvement.

Each surgical modality has its own indications and limitations, and the choice should be based on the specific conditions of the tumor and the patient. In this case, endoscopic-assisted low-temperature plasma resection was selected according to the patient's clinical status: the tumor was hypervascular and located in the tongue base midline, and plasma technology achieved reliable hemostasis, minimal tissue injury, and rapid postoperative recovery. This technique offers advantages including accurate localization, wide field of view, and mild postoperative reactions with prior studies validating its utility in tongue tumors (26). Intraoperative tumor exposure was excellent, with complete resection and negligible bleeding; postoperative tongue motor function was preserved, and no complications (e.g., tongue deviation, numbness) occurred during 2-year follow-up. Pathological confirmation (S-100 and Vimentin positivity) aligned with the diagnostic criteria for schwannomas (15, 27).

This case highlights the diagnostic nuances of rare pediatric tongue root schwannomas and validates endoscopic-assisted low-temperature plasma resection as a safe, effective, child-centered treatment. It aligns with Frontiers in Pediatrics' mission to advance evidence-based care for children and adolescents, offering actionable insights for clinicians managing rare head and neck tumors in this vulnerable population.

## 4 Conclusion

Tongue root schwannoma is an extremely rare tumor in pediatric and adolescent populations. In children and young adolescents, its early clinical symptoms are often nonspecific, and the tumor's appearance may mimic malignant characteristics, easily leading to missed diagnosis or misdiagnosis. Final diagnosis relies on histopathological examination. Surgical excision is the most effective treatment, but pediatric patients require minimally invasive, low-trauma approaches to preserve growth and function. This case describes endoscopic-assisted low-temperature plasma resection for a pediatric tongue root schwannoma, which provides excellent tumor exposure, clear visualization, minimal intraoperative bleeding, and minimal trauma, making it ideal for pediatric patients. Postoperatively, the patient had no complications or recurrence, offering a safe, feasible therapeutic option for this rare pediatric head and neck tumor. This report aligns with the journal's mission to advance evidence-based pediatric care and share innovative, child-centered treatment strategies.

## Data Availability

The original contributions presented in the study are included in the article/Supplementary Material, further inquiries can be directed to the corresponding author.
